# Vaccine Literacy and Social–Cognitive Determinants of Vaccination: Lessons from a Special Issue and Future Directions

**DOI:** 10.3390/vaccines14070552

**Published:** 2026-06-23

**Authors:** Chiara Lorini, Vieri Lastrucci

**Affiliations:** 1 Department of Health Science, University of Florence, Viale GB Morgagni 48, 50134 Florence, Italy; chiara.lorini@unifi.it; 2 Epidemiology Unit, Meyer Children’s Hospital IRCCS, Viale Pieraccini 24, 50139 Florence, Italy

## 1. Introduction

Vaccination remains one of the most effective public health interventions for preventing infectious diseases, reducing morbidity and mortality, and protecting communities across the life course. Nevertheless, the availability of safe and effective vaccines is not, in itself, sufficient to ensure high and equitable uptake. The last decade, and especially the COVID-19 pandemic, has shown that vaccination decisions are shaped by a complex interaction of knowledge, trust, social norms, risk perception, access, previous experiences with healthcare, and exposure to misinformation. The World Health Organization has framed vaccine hesitancy as reluctance or refusal to vaccinate despite vaccine availability and has emphasized the role of confidence, complacency, and convenience in shaping vaccination behavior [1,2]. More recently, the Behavioural and Social Drivers of Vaccination framework has encouraged public health programmes to systematically measure what people think and feel, the social processes that influence them, their motivation, and the practical issues that facilitate or hinder vaccination [3].

Against this background, the Special Issue “Vaccine Literacy and Social–Cognitive Determinants of Vaccination” was conceived to bring together research addressing two closely related domains: vaccine literacy (VL) and the broader social–cognitive determinants of vaccine acceptance, hesitancy, intention, and uptake. The contributions collected in this Special Issue confirm that vaccination behavior cannot be understood through a simple “information deficit” model. Rather, people make vaccination decisions within social contexts, institutional relationships, cultural frameworks, and healthcare systems that may either support or undermine informed and confident choices.

### 1.1. Vaccine Literacy

VL can be understood as a vaccine-specific and relational dimension of health literacy. It refers not only to the ability to access and understand vaccine-related information, but also to the capacity to critically appraise such information, communicate about vaccines, navigate vaccination services, and apply knowledge to make informed decisions for oneself, family members, and the wider community. Earlier approaches to VL have often drawn on the functional, communicative, and critical dimensions of health literacy. Functional VL concerns basic reading, numeracy, and comprehension skills needed to understand vaccine schedules, eligibility criteria, contraindications, and adverse-event information. Communicative or interactive VL includes the ability to discuss vaccines with health professionals, ask questions, express doubts, and use information in everyday interactions. Critical VL involves judging the reliability of information sources, recognizing misleading claims, interpreting uncertainty, and weighing benefits and risks in a given social and epidemiological context [4,5,6].

However, recent conceptual developments suggest that VL should not be reduced to an individual competence or to a proxy for vaccine acceptance. Lorini and colleagues proposed a broader definition based on a scoping review and expert consultation, framing vaccination as a social practice and VL as a multi-level construct encompassing personal, community, population, and organizational perspectives [5]. From this viewpoint, VL includes the knowledge, motivation, and competencies of individuals and communities to access, understand, critically appraise, and apply information about immunization, vaccines, vaccination programmes, and the organizational processes required to access vaccination services. Importantly, this definition does not equate VL with vaccine uptake. Rather, it emphasizes informed decision-making, civic awareness, and the capacity to appreciate the broader population-health impact of vaccination [5].

This perspective is particularly useful for public health because vaccination decisions are rarely purely individual. They are embedded in relationships with family members, peers, healthcare professionals, institutions, media, and communities. Thus, vaccine literacy emerges from the balance between people’s skills and the demands imposed by information systems, services, and social contexts [5].

The increasing complexity of the contemporary information environment makes critical and organizational vaccine literacy especially relevant. Digital platforms have expanded opportunities to access health information, but they have also amplified misinformation, emotionally charged narratives, and conspiracy explanations. Improving VL therefore requires more than producing simplified leaflets or increasing the volume of information. It requires health-literate and vaccine-literate systems able to support trust, transparency, participation, and equitable access. Public health authorities, healthcare organizations, schools, workplaces, community actors, and media systems all contribute to shaping the conditions under which people interpret vaccine information and make vaccination decisions.

At the same time, the relationship between VL and vaccination behavior remains incompletely understood. Evidence suggests that VL may influence vaccine attitudes, intentions, hesitancy, and uptake, but its effect is likely mediated or moderated by confidence, trust, risk perception, social norms, previous experiences, and practical access [6]. This makes VL a crucial, but not sufficient, determinant of vaccination. It should be studied together with the broader social–cognitive determinants of vaccination, particularly when designing interventions aimed at supporting informed, confident, and equitable vaccination choices.

### 1.2. Social–Cognitive Determinants of Vaccination

Vaccination decisions are also shaped by social–cognitive determinants, including beliefs about disease susceptibility and severity, perceived vaccine effectiveness and safety, trust in health professionals and institutions, anticipated regret, perceived social norms, self-efficacy, moral values, conspiracy beliefs, previous experiences, and practical barriers [7]. These factors vary across populations, vaccines, historical moments, and social contexts. For example, determinants of influenza vaccination among healthcare workers may differ from determinants of HPV vaccination among adolescents, COVID-19 vaccination among parents, or routine immunization among migrant communities.

The social–cognitive perspective helps explain why accurate information, although necessary, is often insufficient. Individuals may understand vaccine recommendations and still remain uncertain if they distrust institutions, perceive themselves as being at low risk, fear adverse events, face cultural or language barriers, or encounter logistical obstacles. Conversely, strong recommendations from healthcare professionals, supportive social norms, accessible services, and respectful communication can facilitate vaccination even when knowledge is incomplete. This suggests that VL should be considered in relation to trust, confidence, social belonging, and service responsiveness.

## 2. Overview of Published Papers

The six papers published in this Special Issue contribute to this field by addressing different vaccines, populations, settings, and methodological approaches.

The study conducted among university students in Kazakhstan offers evidence from a middle-income country context. Using the HLS19-VAC instrument, Nukeshtayeva and colleagues examined VL and perceptions among more than 3000 students. Their findings showed generally positive beliefs regarding the importance, safety, effectiveness, and religious compatibility of vaccines, with positive beliefs associated with higher vaccination literacy [8]. This contribution highlights the importance of strengthening VL among young adults, a group that may act both as vaccine decision-makers and as information mediators within families and communities.

A second contribution explored heterogeneity within a childhood-vaccine-hesitant population in Slovenia using latent profile analysis. Rather than treating hesitant individuals as a homogeneous group, Lamot and colleagues identified distinct profiles characterized by different levels of reliance on personal research, overconfidence in knowledge, endorsement of conspiracy theories, use of complementary and alternative medicine, and trust or distrust in the healthcare system [9]. This paper underlines a key implication for public health practice: interventions must be tailored to specific profiles of hesitancy, because a single communication strategy is unlikely to address the needs, beliefs, and motivational structures of all hesitant groups.

The study from Thailand examined parental VL, attitudes toward COVID-19 vaccines, and intention to vaccinate children aged 5–11 years. Maneesriwongul and colleagues found that parental intention was influenced not only by VL, especially its interactive and critical components, but also by attitudes concerning vaccine safety, effectiveness, disease severity, and perceived fatal risks [10]. This contribution is particularly relevant because parents make vaccination decisions on behalf of children, balancing protection, uncertainty, responsibility, and trust in health authorities and professionals.

The Italian study among nursing home staff in Tuscany addressed influenza vaccination intention during the COVID-19 pandemic. Collini and colleagues investigated the relationships between VL, vaccine confidence, and intention to receive influenza vaccination. A central finding was that vaccine confidence mediated the relationship between VL and vaccination intention [11]. This suggests that improving VL alone may not be sufficient to increase uptake if confidence is not simultaneously addressed. For healthcare and long-term care settings, this has direct implications for occupational health policies, staff training, and communication strategies.

Two systematic reviews focused on HPV vaccination and equity. Graci and colleagues examined barriers and facilitators to HPV vaccination among migrant and refugee populations, identifying health literacy, socioeconomic conditions, language barriers, cultural factors, access to information, and trust in health services as crucial determinants [12]. This review highlights the need for inclusive vaccination strategies that are culturally sensitive, linguistically appropriate, and embedded in accessible services. Shen and colleagues investigated HPV vaccine acceptance among men and men with a same-sex orientation, finding suboptimal acceptance and identifying perceived low risk and vaccine cost as important barriers, while recommendations from healthcare professionals and sexual partners acted as facilitators [13]. Together, these reviews show that VL and social–cognitive determinants must be integrated with gender, migration, sexuality, socioeconomic position, and health-system accessibility.

Overall, the papers in this Special Issue offer three main messages. First, VL is a promising construct, but its influence on behavior is mediated and moderated by confidence, trust, attitudes, perceived risk, and context. Second, vaccine hesitancy is heterogeneous; understanding specific profiles and populations is essential for designing tailored interventions. Third, equity must be central: VL cannot be separated from access, language, culture, gender, migration status, and the responsiveness of vaccination services.

## 3. Conclusions

The future research agenda on VL and social–cognitive determinants of vaccination should move in several directions. First, more longitudinal and intervention studies are needed to clarify whether, how, and under what conditions VL leads to vaccine uptake. Cross-sectional studies remain useful for identifying associations, but they cannot fully explain causal pathways. Second, validated and culturally adaptable instruments are needed to compare VL and social–cognitive determinants across countries, vaccines, and population groups. Third, future studies should examine mediation and moderation mechanisms, including the roles of vaccine confidence, institutional trust, misinformation exposure, social norms, and practical access.

Fourth, research should increasingly focus on health-literate vaccination systems. The burden of becoming “vaccine literate” should not rest solely on individuals. Public health authorities, healthcare organizations, schools, workplaces, and community services should reduce unnecessary complexity, provide accessible information, support dialogue, and design services that are easy to navigate. Fifth, interventions should be co-designed with communities, especially those who experience structural barriers or historical mistrust. Migrants, refugees, minority groups, caregivers, healthcare workers, young adults, and groups underserved by existing programmes require strategies that respond to their specific contexts. Particular attention should be given to the identification and engagement of trusted community messengers who can facilitate dialogue, address concerns, and bridge the gap between health systems and underserved populations. Rather than relying exclusively on top-down communication, future interventions should leverage existing community networks and institutions that already enjoy legitimacy and trust. In this context, the development of trained vaccine ambassadors may represent a promising strategy. These individuals should be recruited from trusted community settings—such as community organizations, religious institutions, schools, workplaces, and local associations—and provided with adequate training to communicate evidence-based information, counter misinformation, and support informed vaccination decisions [14].

Finally, the field should bridge VL, behavioral science, and implementation research. Understanding determinants is only the first step; the next challenge is to translate this knowledge into feasible, acceptable, equitable, and sustainable interventions. The contributions in this Special Issue show that vaccination behavior is not only an individual choice, but also the outcome of relationships between people, information, institutions, communities, and systems. Strengthening VL and addressing social–cognitive determinants together may therefore support not only higher vaccine uptake, but also more informed, confident, and equitable vaccination decisions.
